# Expression of Myoferlin in Human Airway Epithelium and Its Role in Cell Adhesion and Zonula Occludens-1 Expression

**DOI:** 10.1371/journal.pone.0040478

**Published:** 2012-07-10

**Authors:** Cleo Leung, Furquan Shaheen, Pascal Bernatchez, Tillie-Louise Hackett

**Affiliations:** 1 The James Hogg Research Centre, Institute for Heart + Lung Health, St Paul’s Hospital, Vancouver, British Columbia, Canada; 2 Department of Anesthesiology, Pharmacology and Therapeutics, University of British Columbia, Vancouver, British Columbia, Canada; Karolinska Institute, Sweden

## Abstract

**Background:**

Normal airway epithelial barrier function is maintained by cell-cell contacts which require the translocation of adhesion proteins at the cell surface, through membrane vesicle trafficking and fusion events. Myoferlin and dysferlin, members of the multiple-C2-domain Ferlin superfamily, have been implicated in membrane fusion processes through the induction of membrane curvature. The objectives of this study were to examine the expression of dysferlin and myoferlin within the human airway and determine the roles of these proteins in airway epithelial homeostasis.

**Methods:**

The expression of dysferlin and myoferlin were evaluated in normal human airway sections by immunohistochemistry, and primary human airway epithelial cells and fibroblasts by immuno blot. Localization of dysferlin and myoferlin in epithelial cells were determined using confocal microscopy. Functional outcomes analyzed included cell adhesion, protein expression, and cell detachment following dysferlin and myoferlin siRNA knock-down, using the human bronchial epithelial cell line, 16HBE.

**Results:**

Primary human airway epithelial cells express both dysferlin and myoferlin whereas fibroblasts isolated from bronchi and the parenchyma only express myoferlin. Expression of dysferlin and myoferlin was further localized within the Golgi, cell cytoplasm and plasma membrane of 16HBE cells using confocal micrscopy. Treatment of 16HBE cells with myoferlin siRNA, but not dysferlin siRNA, resulted in a rounded cell morphology and loss of cell adhesion. This cell shedding following myoferlin knockdown was associated with decreased expression of tight junction molecule, zonula occludens-1 (ZO-1) and increased number of cells positive for apoptotic markers Annexin V and propidium iodide. Cell shedding was not associated with release of the innate inflammatory cytokines IL-6 and IL-8.

**Conclusions/Significance:**

This study demonstrates the heterogeneous expression of myoferlin within epithelial cells and fibroblasts of the respiratory airway. The effect of myoferlin on the expression of ZO-1 in airway epithelial cells indicates its role in membrane fusion events that regulate cell detachment and apoptosis within the airway epithelium.

## Introduction

The airway epithelium forms a continuous, highly regulated physical barrier, which lines the airway lumen, separating the underlying tissue from inhaled environmental antigens. Intercellular epithelial junctions form the structural adhesive forces that maintain the airway epithelial barrier, and are comprised of tight junctions (TJs), adherens junctions (AJs) and desmosomes. AJs mechanically connect adjacent cells and initiate the proliferation and differentiation of cell-cell contacts through homotypic transmembrane E-cadherin adhesions, which are anchored to the actin cytoskeleton and microtubule network by p120 catenin, β-catenin, and α-catenin. TJs are considered the main regulators of paracellular permeability and movement of ions and solutes between cells and are composed of transmembrane proteins such as junction-adhesion-molecules (JAM)s, occludin, and claudins 1–20 which anchor to the cytoskeleton by zonula occludens (ZO)-1, -2, -3 or cingulin. Desmosomes consist of non-classical cadherins that form adhesive bonds between the filament cytoskeleton of epithelial cells, and the lamina propria.

Fusion of cytoplasmic vesicles containing adhesion molecules to the plasma membrane is essential for the formation of selectively permeable membrane barriers. As there is an energy barrier to the fusion process, membrane-fusion events generally require molecules that tether and dock membranes into close proximity of one another (<5–10 nm). Lipid vesicle fusion involves numerous factors, including ferlins, SNAREs and synaptotagmins, to facilitate the fusion of membrane vesicles [1], [2], [3], [4]. In humans (mammals), six ferlin proteins have been identified to date based on their structural similarities and sequence homologies to fer-1; they are dysferlin (Fer1L1) [5], [6], otoferlin (Fer1L2) [7], [8], myoferlin (Fer1L3) [9], [10], Fer1L4 [11], Fer1L5 [12] and Fer1L6 [11]. Each ferlin protein contains a transmembrane domain at the carboxyl terminal which acts as a membrane-anchor and multiple C2 domains, with Ca2+-binding motifs that exhibit a wide range of functions such as phospholipid-binding, signaling and membrane trafficking. The majority of our understanding of mammalian ferlin proteins has come from studies focusing on dysferlin, as mutations in the dysferlin gene lead to limb girdle muscular dystrophy type 2B (LGMD2B) and Miyoshi myopathy [5], [6]. Patients with dysferlinopathy are often diagnosed with defective membrane fusion events in skeletal muscles and the release of surplus vesicles, the contents of which contribute to inflammation and further damage of skeletal muscle [13]. Although functions of myoferlin are less well known, sequencing of the gene revealed that it is highly homologous to dysferlin [9]. In addition, myoferlin has been reported to be responsible for fusion of myoblasts during muscle development and its mutations result in muscle atrophy [14]. Although dysferlin and myoferlin are highly expressed in skeletal and cardiac muscles, studies have confirmed their expression in kidney, placenta, lung, and brain [10], [15], [16], [17], [18]. We have previously demonstrated that dysferlin and myoferlin are present in the caveolae-enriched membrane lipid rafts, isolated from vascular endothelial cells [19], [20]. Importantly, this work also demonstrated that targeted knockdown of dysferlin and myoferlin led to decreased cell proliferation and adhesion through down-regulation of platelet endothelial cell adhesion molecule 1 (PECAM-1) and vascular endothelial growth factor receptor- 2 (VEGFR-2) respectively, in vascular endothelial cells [19], [20]. Similar to the vascular endothelium, the airway epithelium also forms a continuous epithelial sheet on top of a basement membrane. Since dysferlin and myoferlin are membrane repair proteins that are crucial for maintaining membrane integrity, we sort to determine whether these proteins have a functional role in the homeostasis of human airway epithelium. The purpose of this study was thus to examine the expression of dysferlin and myoferlin within the human airway and determine the effects of myoferlin and dysferlin knockdown on airway epithelial functions.

## Materials and Methods

### Subjects and Ethics Statement

Normal human donor lungs not suitable for transplantation and donated for medical research were obtained with written informed consent through the International Institute for the Advancement of Medicine (Edison, NJ). The study was approved by the Providence Health Care Research Ethics Committee of the University of British Columbia; certificate number H0-50110.

### Isolation of Human Airway Epithelial Cells and Lung Fibroblasts

Primary airway epithelial cells were extracted via protease digestion of human airways as previously described [21], [22]. Briefly, bronchi to the 3^rd^ generation were dissected and then rinsed with cold PBS without calcium and magnesium three times to remove blood and mucus. Epithelial layers on intact segments of bronchi (2–4 cm in length) were dissociated at 4°C for 16 hours (h) in 100 ml of MEM (Fisher Scientific) containing 1.4 mg/ml Pronase and 0.1 mg/ml of DNase (Roche Diagnostics). Dissociated epithelial cells were strained through a 70 µm nylon mesh (Becton-Dickison). Cells were then re-suspended and incubated in MEM supplemented with 10%FBS for 10 minutes and washed twice with MEM by centrifugation at 4°C to neutralize the pronase. Cells were then seeded in tissue culture flasks and incubated at 37°C in a humidified 5% CO_2_ atmosphere with bronchial epithelial growth media (Cambrex) until confluent monolayers were formed.

Primary human bronchial and parenchymal fibroblasts were established from the respective bronchial rings and pleura-free lung parenchyma as previously described [23]. Briefly, bronchial and parenchymal tissue from the same lung were dissected into 2 mm^3^ size explants and 4–5 explants placed into the well of 6-well tissue culture plates (BD Biosciences) with 0.75 ml of Dulbecco’s modified Eagle’s medium (DMEM, Invitrogen) containing 10% FBS (Invitrogen), 2 mM L-glutamine and 1% antibiotic/antimycotic solution (Invitrogen) and incubated at 37°C with 95% air and 5% CO_2_. Media was replaced every two days to remove tissue debris and non-adherent cells. After seven days tissue explants were removed and the outgrowth of fibroblasts were maintained until a confluent monolayer was formed. Total protein from both primary epithelial cell and fibroblast cultures was extracted as described under the immunoblot procedures below.

### Cell Lines and Culture Conditions

The bovine aortic endothelial cell (BAEC) and human fetal lung fibroblast (HFL) cell lines were purchased from American Type Culture Collection (ATCC, Manassas, VA, USA). The well described 16HBE14o-, SV40-transformed human bronchial epithelial cell line was obtained from Dr. D. Gruenert (University of Vermont, Burlington, VT, USA) [24]. All cell lines were grown on 6-well tissue culture plates (Corning Costar, Lowell, MA, USA) in DMEM supplemented with 10% fetal bovine serum (FBS) (Hyclone, Logan, USA) and penicillin-streptomycin (Sigma-Aldrich, Oakville, Canada) and cultures were incubated at 37°C in a humidified 5% CO_2_ atmosphere. This study using human cell lines was approved by the Providence Health Care Research Ethics Committee of the University of British Columbia; Certificate number H0-50110-1A.

### Treatment of Small Interfering RNA (siRNA) and Detachment Assay

16HBE cells were grown to 40% confluency in 6-well plates, then transfected with human dysferlin siRNA (5′-CTCCCTGTTTGCGGCCTTCTA-3′), myoferlin siRNA (5′-AACCCTGTCTG GAATGAGATT-3′) or non-silencing scrambled control siRNA at a final concentration of 50, 75 and 100 nM using Oligofectamine in Opti-Mem-1 (Invitrogen, Burlington, ON, Canada) as described by the manufacturer. After 8 hours of transfection, the transfection media was replaced with DMEM containing 10% FBS and penicillin-streptomycin for 72 h after which cells were lysed in protein extraction buffer. Spent cell culture media for each treatment condition was collected to determine the number of detached and dead cells using trypan blue staining and a hemocytometer. To quantify the percentage of cells undergoing apoptosis, we used the flurescein isothiocyanate Annexin V and propidium iodide apoptosis detection kit I as per the manufacturer’s instructions (BD Pharmingen) and cells were analyzed immediately by flow cytometry using a BD LSRII machine and flow cytometry analysis was performed using DiVa software.

### Immunoblot

Proteins were extracted from cell monolayers using protein extraction buffer with protease and phosphatase inhibitor cocktails (Sigma). The total protein concentration of each cell lysate was determined using the DC protein assay as instructed by the manufacturer (Biorad). Equal amounts of protein lysates (80 µg) were loaded to resolve the proteins by sodium dodecyl sulfate-polyacrylamide gel electrophoresis and then transferred to nitrocellulose membranes. The primary antibodies used for immuno blot were dysferlin (NCL-Hamlet, Novocastra), myoferlin (ab76746, Abcam), E-cadherin (sc-8426, Santa Cruz), caveolin-1 (sc-894, Santa Cruz), β-tubulin (16–231, Upstate), VEGFR-2 (sc-504, Santa Cruz), ZO-1(ab59720, Abcam), claudin-1 (37–4900, Life Technologies Corporation), occludin (71–1500, Invitrogen), fibronectin-EDA (MAB1940, Chemicon International, Temecula, CA) and HSP90 (610418, BD Biosciences). Detection was performed with IR700 anti-mouse and IR800 anti-rabbit antibodies (Cell Signaling Technology) and the Odyssey Infrared Imaging System (LI-COR Biotechnology, Lincoln, Nebraska) using a manufacturer’s protocol. Density of the bands was analyzed with Odyssey software 1.1 (LI-COR Biotechnology) using two infrared channels independently. The results are expressed as protein of interest/β-tubulin or HSP-90 density ratio.

### Immunohistochemical Staining

Donor airway sections (5 µm) were deparaffinized, rehydrated and antigens were retrieved by autoclaving the sections in citrate target retrieval solution (Dako) for 15 minutes at 120°C and 30 psi. Endogenous peroxidase was quenched with 3% hydrogen peroxide for 20 minutes and non-specific binding was blocked with 10% horse serum. Slides were incubated overnight with antibodies against either human dysferlin (1∶40, clone Ham1/7B6, NCL-Hamlet, Novacastra) or myoferlin (1∶500, clone 7D6, ab76746, Abcam), ZO-1 (1∶200 at 4°C in 5% horse serum. Subsequent to three washes in Tris-buffered saline (TBS), sections were then incubated with horse anti-mouse secondary antibody (1∶100, Vector Labs) for 2 h, followed by incubation with streptavidin-horse radish peroxidase (Dako) for 20 minutes. Staining was visualized using brown chromogen 3,3-diaminobenzidine (Dako) and counterstained with hematoxylin (Sigma). Slides were then dehydrated and mounted with Cytoseal 60 (Richard-Allan Scientific). Negative controls were performed in parallel with mouse IgG isotype control (sc-2025, Santa Cruz) and by omitting the primary antibody.

### Immunofluorescent Staining and Confocal Microscopy

16HBE cells were seeded on chamber slides (BD Falcon, NJ, USA), washed in phosphate-buffered saline (PBS, Sigma-Aldrich) pH 7.4, fixed in 4% paraformaldehyde (Fisher Scientific, Ottawa, ON, Canada) for 20 minutes and blocked with 10% goat serum (Gibco-Invitrogen) for 20 minutes at room temperature (RT). Cells were stained with either 1∶40 dysferlin or 1∶500 myoferlin and 1∶200 ZO-1 or 1∶200 GM130 (610822, BD) antibodies in PBS with 0.1% saponin overnight at 4°C. After removing the primary antibodies and washing the cells with PBS for 3 times at RT, secondary antibody conjugated with 10 µg/ml goat anti-mouse IgG Alexa Fluor 594 (Invitrogen) was added and incubated for 2 h at RT. Cells were washed again with PBS and then incubated with 1 µg/ml of 4,6-diamidino-2-phenylindole (DAPI) before visualizing with a Nikon fluorescent microscope incorporated with a C-spot camera (Nikon Instruments). Fluorescent images were captured using a Leica AOBS SP2 laser scanning confocal microscope (Leica) with Zeiss LSM 510 software, version 3.2 and analyzed with Volocity software (Improvision).

### Statistical Analysis

Quantitative data are presented as mean ± SEM of three independent experiments. One-way analyses of variance with Bonferroni’s post-hoc correction were used to compare between the treatment groups. P values of less than 0.05 were considered statistically significant. All statistical analyses were performed using Prism Version 5.0 software (Graphpad, Software Inc.).

## Results

### Expression of Myoferlin and Dysferlin within the Human Airway

**Figure 1:**
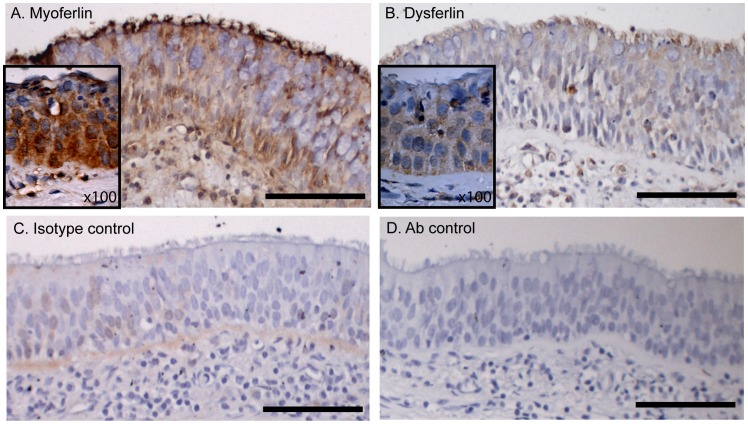
Dysferlin and myoferlin expression in normal human airway epithelium. Airway sections were obtained from non-diseased human subjects (n = 5) and sections were stained with (A) myoferlin, (B) dysferlin, (C) isotype control and (D) secondary antibodies. The images are representative of the five individuals studied. Scale bar = 100 µM, insert image ×100 magnification.

To study the expression of myoferlin and dysferlin within the human airway, we performed immunohistochemical staining. As shown in the representative images of human airway, myoferlin and dysferlin (*brown*) are expressed in both basal and ciliated airway epithelial cells in addition to cells within the lamina propria, which consists mainly of fibroblasts and infiltrating inflammatory cells (Figure 1A and B, respectively). Staining with mouse immunoglobulin control antibody at the same concentration did demonstrate minor non-specific staining (Figure 1C) whereas the secondary antibody control alone produced no non-specific staining (Figure 1D).

### Expression of Dysferlin and Myoferlin in Human Lung Cells in vitro

**Figure 2:**
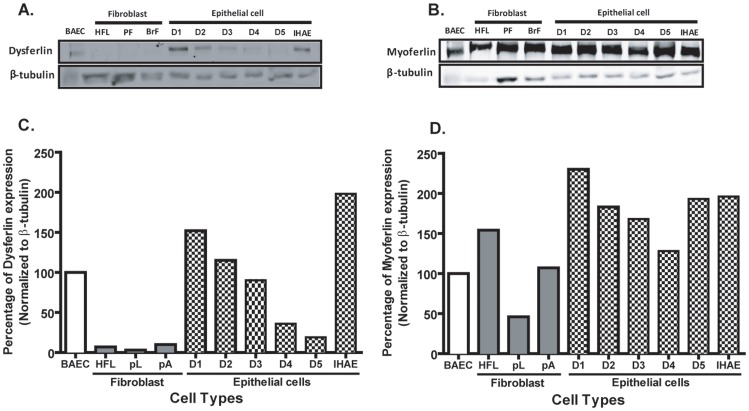
Dysferlin and myoferlin expression in human airway epithelial cells and fibroblasts. Total cell lysates from primary human lung parenchymal fibroblasts (PF), bronchial fibroblast (BrF), airway epithelial cells derived from normal donor lung tissues (D1–D5), bovine aortic endothelial cell (BAEC), human fetal lung fibroblasts (HFL) and bronchial epithelial cell (16HBE) lines were analyzed for the expression of (A) dysferlin and (B) myoferlin by immunoblot. The band intensity of (C) dysferlin and (D) myoferlin was normalized to β-tubulin and compared to BAEC to quantify the expression levels of the ferlin proteins by densitometry.

We further confirmed our findings using immunohistochemistry by evaluating the expression of dysferlin and myoferlin by immunoblot in both cell lines and primary cells isolated from human lung. All cells were grown in normal growth conditions to confluency and total protein was isolated to analyze dysferlin and myoferlin expression. Figure 2A shows dysferlin was expressed in both primary airway epithelial cells from five healthy donor lungs and the airway epithelial cell line 16HBE, but not in primary bronchial, lung or cell line fibroblasts. In comparison, all airway epithelial cells and fibroblasts express myoferlin at similar expression levels to that found in bovine aorta endothelial cells (BAECs), which we have previously reported to express both myoferlin and dysferlin (Figure 2B). To demonstrate the expression of dysferlin and myoferlin in each cell type the densitometry values normalized to loading control β-tubulin are expressed as a percentage of expression in BAEC (Figure 2C and D).

### Localization of Dysferlin and Myoferlin Expression in Airway Epithelial Cells

**Figure 3:**
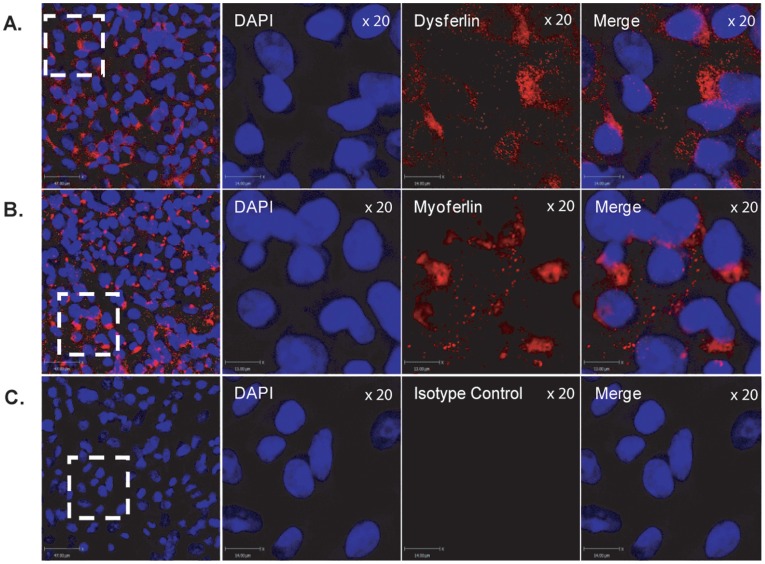
Localization of dysferlin and myoferlin in airway epithelial cells. 16HBE cells were grown to confluency on 8 well chamber slides and fixed for immunofluorescence analysis. Immunofluorescent staining for (A) dysferlin (*red*), (B) myoferlin (*red*), (C) mouse immunoglobulin isotype, and nuclei stained with 4′,6-diamidino-2-phenylindole (*blue*), were used to examine the localization of dysferlin and myoferlin. Scale bars are equal to 47 µm, and 14 µm on the digital zoomed images.

To localize the expression of dysferlin and myoferlin in airway epithelial cells, confluent monolayers of 16HBE cells were analyzed by confocal microscopy using immunofluorescent staining. At low magnification, dysferlin and myoferlin (red staining) were found to be expressed abundantly in 16HBE cells in monolayer culture as demonstrated in Figure 3 panels A and B compared to the isotype control (Figure 3, panel C). Using a digital zoom (white boxed areas), dysferlin and myoferlin was observed to be expressed within the cell cytoplasm and cell membrane but not within the nucleus as identified by DAPI (blue straining, Figure 3, panels A & B), compared to the isotype control antibody (Figure 3, panel C). We also observed dysferlin and myoferlin (red staining) expression within Golgi membranes by co-localization with the Golgi matrix protein, GM-130 (green staining), as shown in figure S1A and B.

### Down-regulation of Dysferlin and Myoferlin by Gene Silencing in Airway Epithelial Cells

**Figure 4:**
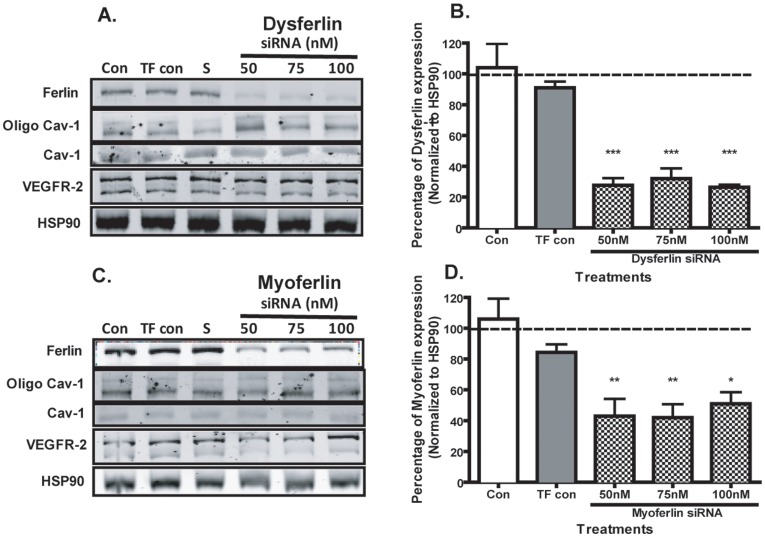
No effect of dysferlin or myoferlin knockdown on VEGFR-2 and caveolin-1 expression. 16HBE cells were transfected with oligofectamine transfection reagent (TF con), scrambled siRNA (S), dysferlin or myoferlin siRNA at 50 nM, 75 nM and 100 nM for 72 h. The representative immunoblot of three independent experiments demonstrates that siRNA specific for (A) dysferlin and (C) myoferlin had no effect on caveolin-1 or vascular endothelial growth receptor 2 (VEGFR-2) protein expression. Densitometry analysis of band intensities for (B) dysferlin and (D) myoferlin normalized to HSP90 compared to the scrambled siRNA control (dotted line), indicate knock down of 50% or more for each ferlin protein. Values are represented as mean (± SEM) of three independent experiments. A significant difference between the treatment groups with scrambled siRNA is indicated by asterisk (*p<0.05; **p<0.01, ***p<0.005).

To identify the potential roles of dysferlin and myoferlin in airway epithelial cells, previously designed dysferlin and myoferlin specific siRNA’s were used to knock down protein expression [20], [25]. 16HBE cells were transfected with siRNA sequences specific to dysferlin, myoferlin and a scrambled siRNA control at concentrations of 50 nM, 75 nM and 100 nM for 72 h. As demonstrated by the representative immunoblot in Figure 4A and the densitometry analysis normalized to HSP90 in Figure 4B, dysferlin specific siRNA significantly knocked down dysferlin expression by 72% at 50 nM, 68% at 75 nM and 74% at 100 nM compared to the non-targeting scrambled siRNA control. Likewise, myoferlin siRNA also significantly knocked down myoferlin expression by 57% at 50 nM, 58% at 75 nM and 49% at 100 nM compared to the scrambled siRNA control (Figure 4C and D).

### Silencing of Dysferlin and Myoferlin Genes does not Change the Expressions of Caveolin-1 and VEGFR-2

Silencing of dysferlin and myoferlin genes may invoke a compensatory response to resume normal protein expression of dysferlin and myoferlin. Caveolin-1 has been shown to regulate the expression of dysferlin and interact with myoferlin in membrane resealing events [26], [27]. Airway epithelial cells express high levels of caveolin-1, however as demonstrated by the representative immunoblots we observed no change in caveolin-1 monomer or oligomer expression in 16HBE cells treated with dysferlin or myoferlin siRNA (Figure 4A & C, respectively).

Furthermore we have previously demonstrated that knockdown of myoferlin, but not dysferlin, leads to reduced expression of vascular endothelial growth factor receptor 2 (VEGFR-2) in BAEC [20], [25]. However in 16HBE cells, we observed no changes in VEGFR-2 expression following myoferlin or dysferlin knockdown indicating these ferlin proteins may have cell specific roles in membrane fusion and trafficking events.

### Knockdown of Myoferlin Changes Airway Epithelial Cell Morphology and Cell Detachment

**Figure 5:**
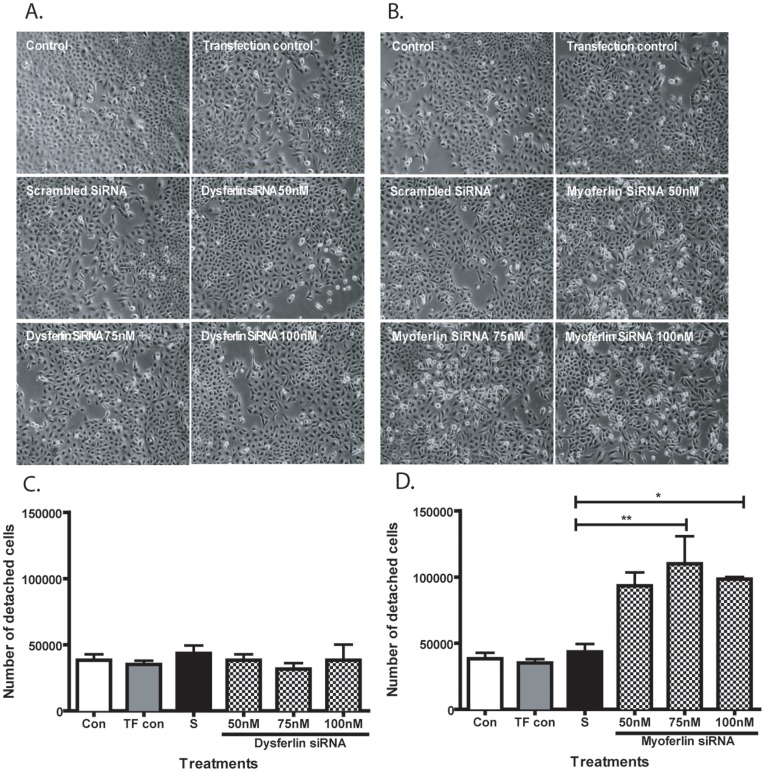
Knockdown of myoferlin but not dysferlin alters cell morphology and cell detachment. Phase contrast images captured at 10× magnification of 16HBE cells treated with control media, transfection control, scrambled siRNA, and (A) dysferlin siRNA or (B) myoferlin siRNA at 50 nM, 75 nM and 100 nM for 72 h. Detached cells within cell culture supernatants were counted in each treatment group following (C) dysferlin or (D) myoferlin siRNA treatment using a hemocytometer. Data are presented as mean ± SEM of three independent experiments. A significant difference between the treatment groups and scrambled siRNA control is indicated by asterisk (*p<0.05; **p<0.01).

We next examined the effects of down-regulating dysferlin and myoferlin using siRNA transfection on cell morphology and attachment. As demonstrated in the phase contrast images of 16HBE cell monolayers (Figure 5A) following 72 h of dysferlin siRNA transfection, we observed no significant changes in cell morphology. In contrast, knockdown of myoferlin led to a loss of normal epithelial cuboidal morphology to a spindle-shaped phenotype at all siRNA concentrations and increased numbers of rounded and detached cells (Figure 5B). To verify our observations of cell detachment following 72 h of siRNA treatment, the cell culture media was isolated and cell counts conducted. We found no significant difference between the number of detached 16HBE cells treated with dysferlin siRNA and the scrambled siRNA control (Figure 5C). In comparison, cells treated with myoferlin siRNA at 75 nM and 100 nM concentrations showed significantly greater numbers of detached 16HBE cells compared to the scrambled siRNA control (Figure 5D).

### Myoferlin Knockdown Induces Airway Epithelial Cell Detachment Resulting in Cell Death but not Inflammation

**Figure 6:**
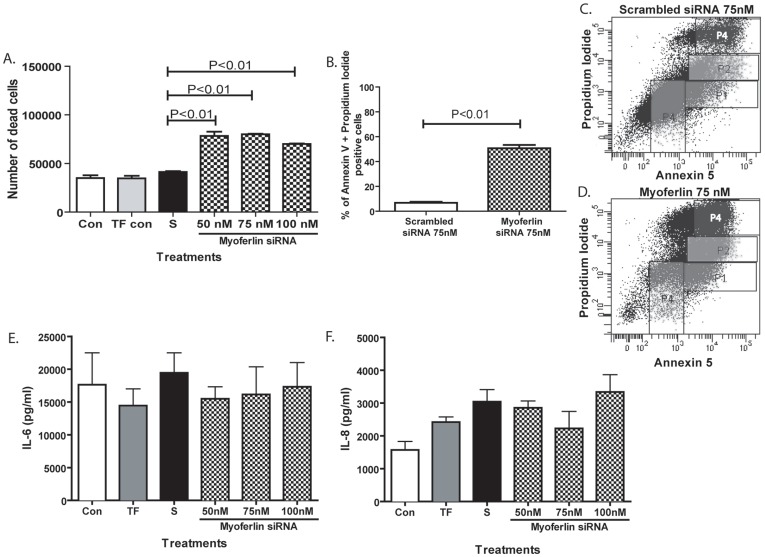
Myoferlin knockdown induces changes in cell detachment and apoptosis but not inflammation. 16HBE cells were treated with control media (con), transfection control (TF con), scrambled siRNA (S), or myoferlin siRNA at 50 nM, 75 nM and 100 nM for 72 h. Cell culture supernatants were removed and analyzed for (A) dead cells using trypan blue assay and (B) percentage of cells positive for annexin 5 and propidium iodide by FACs analysis, which are surrogate markers of apoptosis. Representative FACs plots of 16HBE cells treated with (C) scrambled siRNA control compared to (D) myoferlin siRNA at 75 nM concentration indicate elevated numbers of cells positive for propidium iodide and annexin 5 (gated in P4). Cell culture supernatants were also analyzed for inflammatory cytokines (E) interleukin (IL)-6 and (F) IL-8. Data are presented as mean ± SEM of six independent experiments. A significant difference of *p*<0.05 was considered significant.

As the changes in cell morphology and cell detachment observed in cells treated with myoferlin siRNA could be an indication of cell death, we evaluated the viability of detached cells within the culture media using trypan blue dye. We found that the number of dead cells following myoferin knockdown did increase at all siRNA concentrations (Figure 6A). Cell apoptosis was evaluated after 72 h of myoferlin siRNA treatment by flow cytometry analysis using Annexin-V and propidium iodide (PI) staining (Figure 6B). During the early stages of apoptosis, translocation of phosphatidylserine from the inner to outer plasma membrane can be detected using Annexin-V and PI staining. The results show that the percentage of apoptosis was 12.3% in cells treated with 75 nM scrambled siRNA control compared to 46.8% in 16HBE cells treated with 75 nM myoferlin siRNA (Figures 6C and D, respectively). We next examined if knockdown of myoferlin resulted in elevated release of innate pro-inflammatory cytokines interleukin (IL)-6 and IL-8. As demonstrated in Figure 6E and 6F, the concentration of IL-6 and IL-8 released by 16HBE cells did not alter following treatment of myoferlin specific siRNA compared to transfection and scrambled siRNA controls.

### Myoferlin Knockdown in Airway Epithelial Cells Results in Loss of ZO-1 Expression

**Figure 7:**
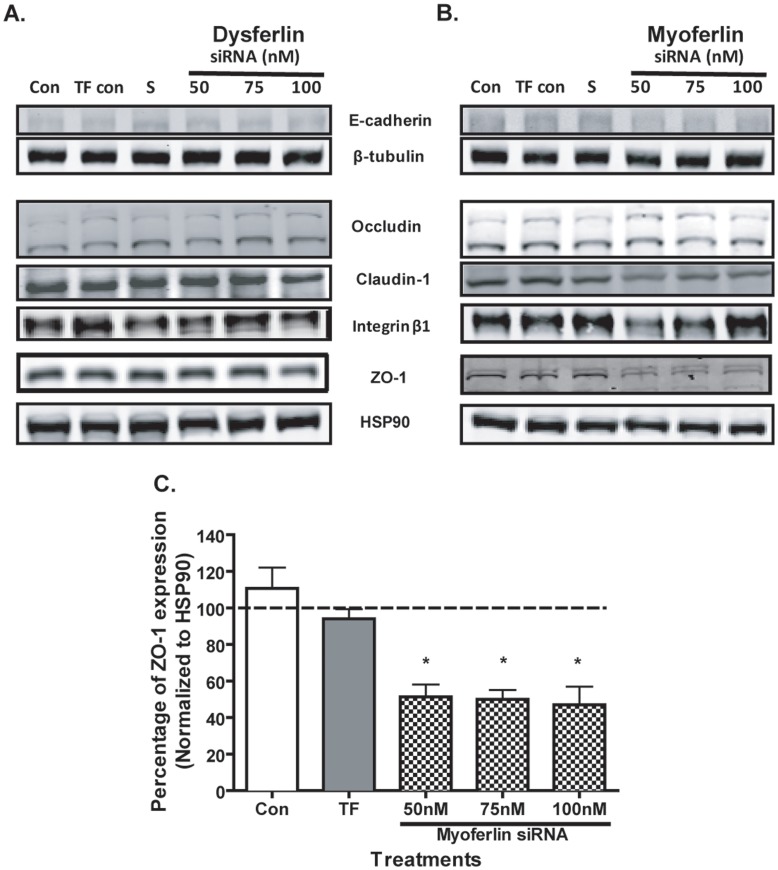
Myoferlin knockdown decreases the expression of tight junction molecule ZO-1. 16HBE cells were treated with control media (con), transfection control (TF con), scrambled siRNA (S), and (A) dysferlin or (B) myoferlin siRNA at 50 nM, 75 nM and 100 nM for 72 h. Total cell lysates were prepared to analyze the expressions of adherens junction molecule E-cadherin and tight junction molecules occludin, claudin 1, zonula occludens-1 (ZO-1) and integrin β1 as demonstrated by the representative immuno blot from three independent experiments. β-tubulin and HSP90 served as loading controls. (C) Densitometry analysis of band intensities for ZO-1 normalized to HSP90 compared to the scrambled siRNA control (dotted line). Values are represented as mean (± SEM) of three independent experiments. A significant difference between the treatment groups and scrambled siRNA control is indicated by asterisk (*p<0.05).

We hypothesized that the morphological changes and cell detachment observed in epithelial cells treated with myoferlin siRNA may indicate loss of cell adhesion. To test this hypothesis, we evaluated the expression of adherens junction molecule, E-cadherin, as well as tight junction molecules occuldin, claudin-1, ZO-1 and extracellular matrix (ECM) adhesion receptor integrin β1 during knockdown of dysferlin and myoferlin. Dysferlin knockdown did not result in cell detachment; and as expected, we did not detect any difference in the expression of any of the adhesion proteins measured (Figure 7A). In comparison, myoferlin knockdown led to a decrease in the expression of ZO-1 at all myoferlin siRNA concentrations, but not E-cadherin, occludin, claudin-1 or integrin β1 when compared to the transfection control conditions (Figure 7B and C). We and others have previously shown that loss of cell-cell contacts and acquisition of a spindle shape morphology are two of the initial morphological changes associated with epithelial-mesenchymal transition (EMT). To confirm that loss of ZO-1 was not associated with EMT we analyzed the expression of E-cadherin and mesenchymal marker fibronectin-EDA by immuno blot. As shown by the densitometry analysis in figure S2 we observed no differences in the expression of E-cadherin or fibronectin-EDA.

### Myoferlin and ZO-1 Proteins Co-localize in Airway Epithelial Cells

**Figure 8:**
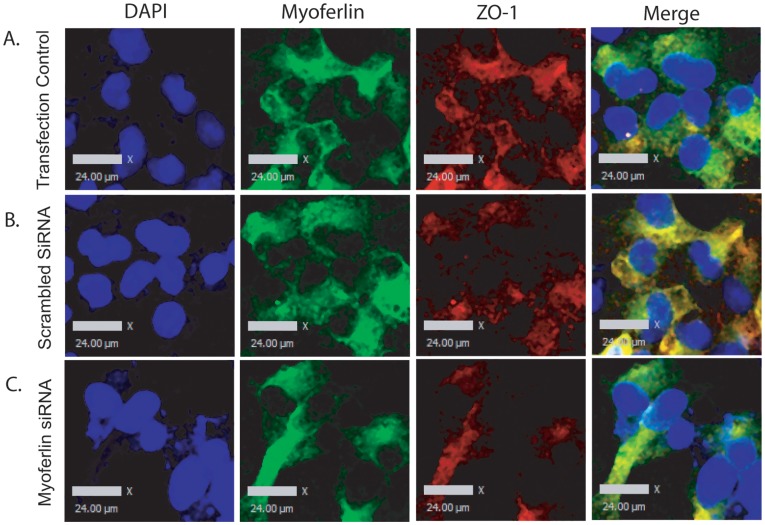
Co-localization of myoferlin and ZO-1. 16HBE cells were grown on 8 well chamber slides and treated with (A) transfection control, (B) scrambled siRNA, or (C) myoferlin siRNA both at 75 nM concentration for 72 h then fixed for immunofluorescence analysis. Immunofluorescent staining for myoferlin (*green*), zonula occludens 1 (*red*) and nuclei stained with 4′,6-diamidino-2-phenylindole (*blue*); were used to examine the localization of ZO-1 and myoferlin following siRNA treatments. Scale bars are equal to 24 µm on all images.

To understand the association between myoferlin and ZO-1, we looked at the distribution patterns of myoferlin and ZO-1 in 16HBE cells using confocal microscopy. The distribution pattern of ZO-1 staining (*green*) was co-localized to that of myoferlin staining (*red*) as evidenced by the overlay images, which demonstrate the two co-localized proteins by yellow staining (Figure 8). Furthermore, when myoferlin expression was knocked down using siRNA (75 nM), we observed reduced expression of both myoferlin and ZO-1 as demonstrated by loss of total (*green and red*) and co-localized staining (*yellow*) compared to the transfection control conditions.

## Discussion

Under normal circumstances, the polarized airway epithelium forms a highly regulated and semi-permeable barrier through the formation of cell-cell adhesions. The formation of these cell-cell adhesion junctions requires the coordinated fusion and exocytosis of vesicles containing adhesion molecules at targeted areas within the cell plasma membrane. In this study, we demonstrate for the first time that membrane fusion proteins of the ferlin family, dysferlin and myoferlin, are expressed within airway epithelial cells of the human lung. Furthermore, we demonstrate that down-regulation of myoferlin, but not dysferlin using siRNA, results in loss of epithelial tight junction protein ZO-1 expression, cell detachment and apoptosis.

Sorting of cell membrane-associated proteins occurs predominantly in the *trans*-Golgi network in both polarized epithelia and “non-polarized” fibroblasts through well-defined cytoplasmic domain-sorting signals [28], [29], [30] and specialized organization of the actin and microtubule cytoskeleton beneath the plasma membrane [31]. During membrane fusion, a membrane bound structure fuses with the plasma membrane and expels its contents to the exterior. The fusion mechanisms involved include aggregation of the membranes, resulting in close bilayer apposition; destabilization and rupture of the membrane; and merging of the aqueous contents and membrane components [32]. This fusion of vesicles within a target plasma membrane is regulated by organelle-specific Rab GTPases, vesicle-tethering complexes [33] and SNAREs [34]. Ferlin family members are known to mediate membrane fusion events upon membrane damage, endocytosis and exocytosis [27], [35]. Ferlins anchor to the plasma membrane via a single C-terminal transmembrane domain and contain C2 domains that mediate lipid and protein binding. Both dysferlin and myoferlin contain C2 domains which can coordinate calcium ions within a negatively charged binding pocket to regulate calcium-activated events, akin to the well characterized classical mediators of vesicle fusion, the synaptotagmins, which contain two C2 domains [36], [37].

The roles of dysferlin and myoferlin in membrane fusion events have primarily focused on skeletal muscles, as mutations particularly in the dysferlin gene are associated with the development of muscular dystrophy, a muscle wasting disease. Since then, dysferlin and myoferlin have also been demonstrated to be expressed in a number of other cell types including myoblasts, inflammatory cells, endothelial cells and specialized epithelium of the placenta, renal tubular and glomerular structures [16], [38]. Here, we report that dysferlin and myoferlin are expressed in the airway epithelium at similar levels to vascular endothelial cells whereas bronchial and lung fibroblasts only express myoferlin. We observed that the sub-cellular distribution of dysferlin and myoferlin within airway epithelial cells is localized to the Golgi-membrane, cytoplasm and plasma membrane. Furthermore, this staining was punctate in appearance, suggesting expression was localized to vesicles. Previous studies have reported low expression levels of dysferlin and myoferlin in human lung [10], [15], but these studies did not investigate the expression of these proteins in specific cell types.

To determine the function of dysferlin and myoferlin within the airway epithelial cells, we used specific siRNAs to knock down protein expression. Down-regulation of myoferlin, but not dysferlin, resulted in loss of cuboidal epithelium and acquisition of a spindle-shape morphology. In addition, the numbers of detached cells within cultures also significantly increased following myoferlin knockdown when compared to dysferlin and control conditions. We have previously demonstrated dysferlin knockdown impairs the proliferation, migration and adhesion of sub-confluent vascular endothelial cells through loss of the adhesion molecule, PECAM-1 [19]. Whereas myoferlin knock down led to a decrease in vascular endothelial cell proliferation, and migration that was associated with decreased expression of VEGFR-2, the receptor of vascular endothelial growth factor, an important molecule in vasculogenesis and angiogenesis [20]. In addition dysferlin and myoferlin have been shown to interact with caveoli-1n in lipid rafts of endothelial cells, skeletal muscle and fibroblasts [27], [39]. As PECAM-1 is not expressed on epithelial cells, we quantified the expression of VEGFR-2 and caveolin-1 in airway epithelial cells. We expected that knockdown of dysferlin and myoferlin may stimulate a compensatory response on caveolin-1 in airway epithelial cells. However, we did not see any difference in the expression of caveolin-1, suggesting that dysferlin and myoferlin have tissue or cell-specific functions. Interestingly, although we observed that down-regulation of myoferlin in airway epithelial cells was not associated with VEGFR-2 expression, it was associated with the loss of TJ protein, ZO-1. This finding further suggests that dysferlin and myoferlin may have different regulatory roles of membrane-associated proteins in different tissues. Interestingly, myoferlin associated down-regulation of ZO-1 and cell detachment was not associated with expression changes in the other transmembrane TJ and AJ proteins analyzed, which included occludin, claudin 1 and E-cadherin. In support of our findings, it has recently been shown in the mouse jejunum epithelium that the process of cell shedding requires redistribution of ZO-1 prior to cell extrusion and that shed epithelial cells undergo nuclear chromatin condensation, a hallmark of apoptosis [40]. This study and others suggest that ZO-1 rearranges beneath dividing and shedding cells to maintain epithelial barrier function by connecting neighboring cells surrounding an extruding cell [41], [42], [43], [44]. Thus, loss of ZO-1 expression via down-regulation of myoferlin expression may be an active participant in catalyzing cell extrusion. This would support our findings of increased numbers of epithelial cells positive for annexin V and PI staining, which are markers for apoptosis, within cultures treated with myoferlin siRNA. As calcium-dependent intercellular adhesion is required for ZO-1 localization to the TJ [45], [46], we propose that in epithelial homeostasis, the multiple C2-calcium binding domains of myoferlin may play an important role in regulating the graduated fusion in response to gradients of calcium release, for vesicle trafficking. In support of our hypothesis, recent studies have shown that myoferlin forms a protein complex with dynamin 2 (Dyn-2) that is essential for the fission of endocytic vesicles [47], [48] and that tight junctional proteins mislocalize and cell polarity is lost upon Dyn-2 depletion, suggesting a dynamin-myoferlin mediated endocytic pathway maybe involved in the spatial regulation and distribution of tight junction proteins and their binding partners.

Tight junctions are responsible for regulating paracellular permeability and maintaining cell polarity, which are often referred to as “barrier” and “fence” function, respectively. TJs also play a role in signaling pathways involved in epithelial proliferation, gene expression and differentiation [49], [50]. ZO-1 via its SH3 domain, binds ZONAB (ZO-1-associated nucleic-acid-binding protein) a Y-box transcription factor, sequestering it in the cytoplasm and inhibiting its transcriptional activity [49], [50]. Following disassembly of ZO-1 from the tight junction, ZONAB is able to interact with the cell cycle kinase cdk4 to regulate the transcription of cell cycle genes including cyclin D1 and PCNA, which lead to the proliferation of epithelial cells in culture [51], [52], [53]. ZO-1 expression has been shown to be localized both at the cell membrane, within the cell cytoplasm and nucleus in dividing cells. These data support our co-localization of ZO-1 and myoferlin by confocal microscopy as both proteins were found with the cytoplasm and cell membrane. Myoferlin has been shown previously to be ubiquitously expressed in developing skeletal muscle, cardiac muscle, placenta [10] and caveolae-enriched buoyant lipid rafts in endothelial cells [20]. Interestingly, myoferlin shows developmental ‘switching’ in skeletal muscle as it is highly expressed in proliferating mononuclear cells and becomes downregulated with myogenic maturation [54], [55]. In contrast, dysferlin is expressed at low levels in proliferative myoblasts, and becomes robustly expressed in fused myotubes and in mature muscle [54]. This suggests that myoferlin and dysferlin may possess both overlapping and specialized roles, with functional specialization related to distinct requirements of proliferative and differentiated cells. In our study, we found that myoferlin and to a limited extent dysferlin was expressed ubiquitously within basal, ciliated and mucus expressing cells within normal human airway epithelium. However our study does not investigate the expression and localization of myoferlin and dysferlin following epithelial proliferation or repair or the compensatory responses by other multiple C2 domain protein family members, in the absence of either protein. Future studies in our laboratory are aimed at unraveling the mechanisms by which myoferlin regulates ZO-1 expression during epithelial damage.

In conclusion, our study provides evidence that dysferlin and myoferlin are present in several cell types within the human airway. Furthermore, down-regulation of myoferlin in airway epithelial cells triggers loss of ZO-1 expression, cell shedding and apoptosis. These *in vivo* and *in vitro* data thus demonstrate that myoferlin in airway epithelial cells may play a role in maintaining epithelial homeostasis through trafficking of tight junction protein ZO-1. Future studies are required to understand the potential roles of myoferlin during epithelial repair.

## Supporting Information

Figure S1 **Localization of dysferlin and myoferlin with GM130 in airway epithelial cells.** 16HBE cells were grown to confluency on 8 well chamber slides and fixed for immunofluorescence analysis. Immunofluorescent staining for (A) dysferlin (*red*), (B) myoferlin (*red*), Golgi membrane marker GM130 (green), and nuclei stained with 4′,6-diamidino-2-phenylindole (*blue*) were used to examine the localization of dysferlin and myoferlin within the Golgi membrane. Scale bars are equal to 20 µm.
(TIF)

Figure S2 **Myoferlin knockdown does not induce features of epithelial-mesenchymal transition.** 16HBE cells were treated with control media (con), transfection control (TF con), scrambled siRNA (S), and (A) myoferlin siRNA at 50 nM, 75 nM and 100 nM for 72 hours. Total cell lysates were prepared to analyze the expressions of adherens junction molecule E-cadherin and fibronectin-EDA as demonstrated by the representative immuno blot from three independent experiments. HSP90 served as a loading control. (B) Densitometry analysis of band intensities for E-cadherin and (C) fibronectin-EDA normalized to HSP90 compared to the scrambled siRNA control (dotted line). Values are represented as mean (± SEM) of three independent experiments.
(TIF)
