# Supplementary figures and images for: Expression of Myoferlin in Human Airway Epithelium and Its Role in Cell Adhesion and Zonula Occludens-1 Expression
Source: PLoS One. 2012 Jul 10;7(7):e40478. doi: 10.1371/journal.pone.0040478 (PMC3393691; doi:10.1371/journal.pone.0040478)

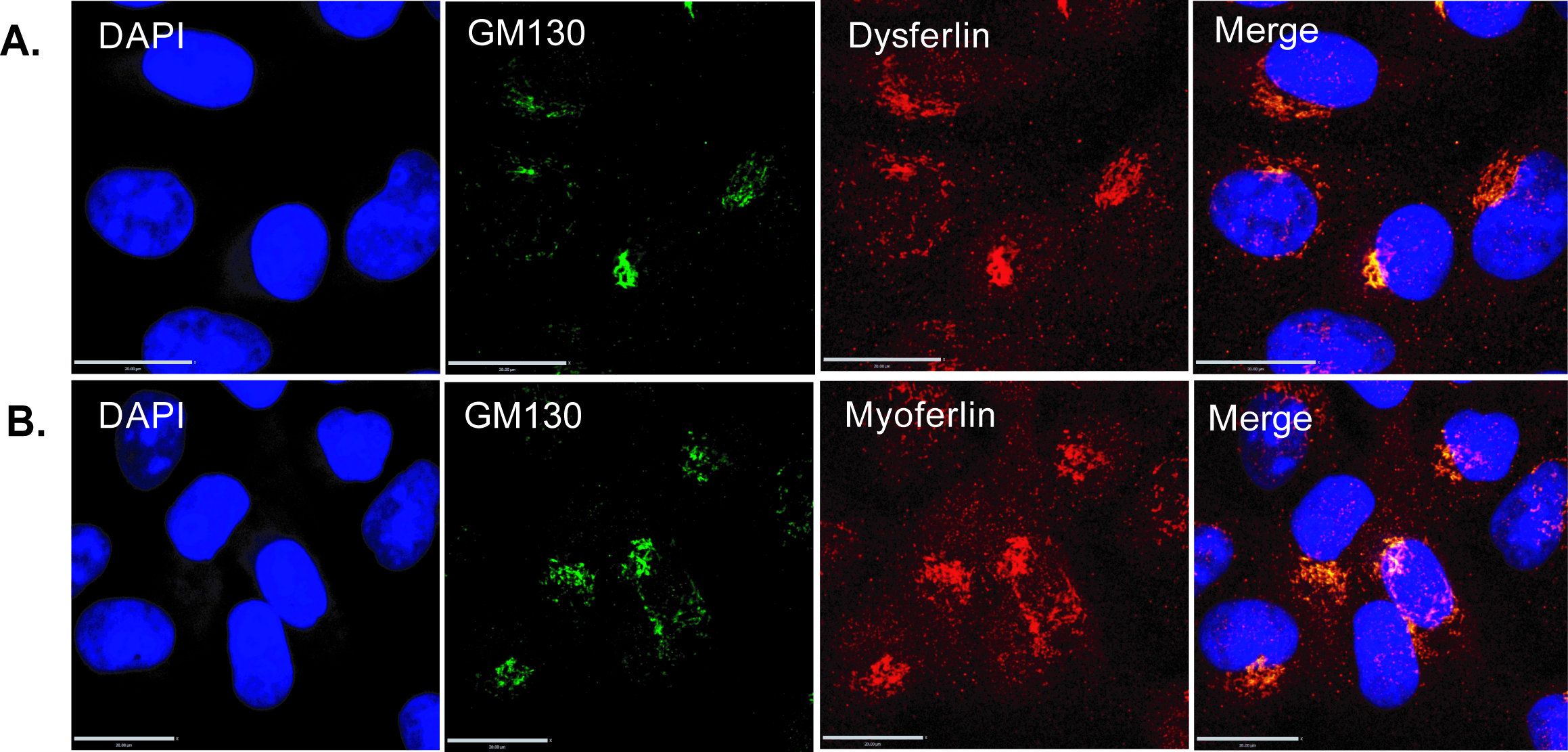

Supplement: Figure S1 — Localization of dysferlin and myoferlin with GM130 in airway epithelial cells. 16HBE cells were grown to confluency on 8 well chamber slides and fixed for immunofluorescence analysis. Immunofluorescent staining for (A) dysferlin (red), (B) myoferlin (red), Golgi membrane marker GM130 (green), and nuclei stained with 4′,6-diamidino-2-phenylindole (blue) were used to examine the localization of dysferlin and myoferlin within the Golgi membrane. Scale bars are equal to 20 µm. (TIF) [file pone.0040478.s001.tif]

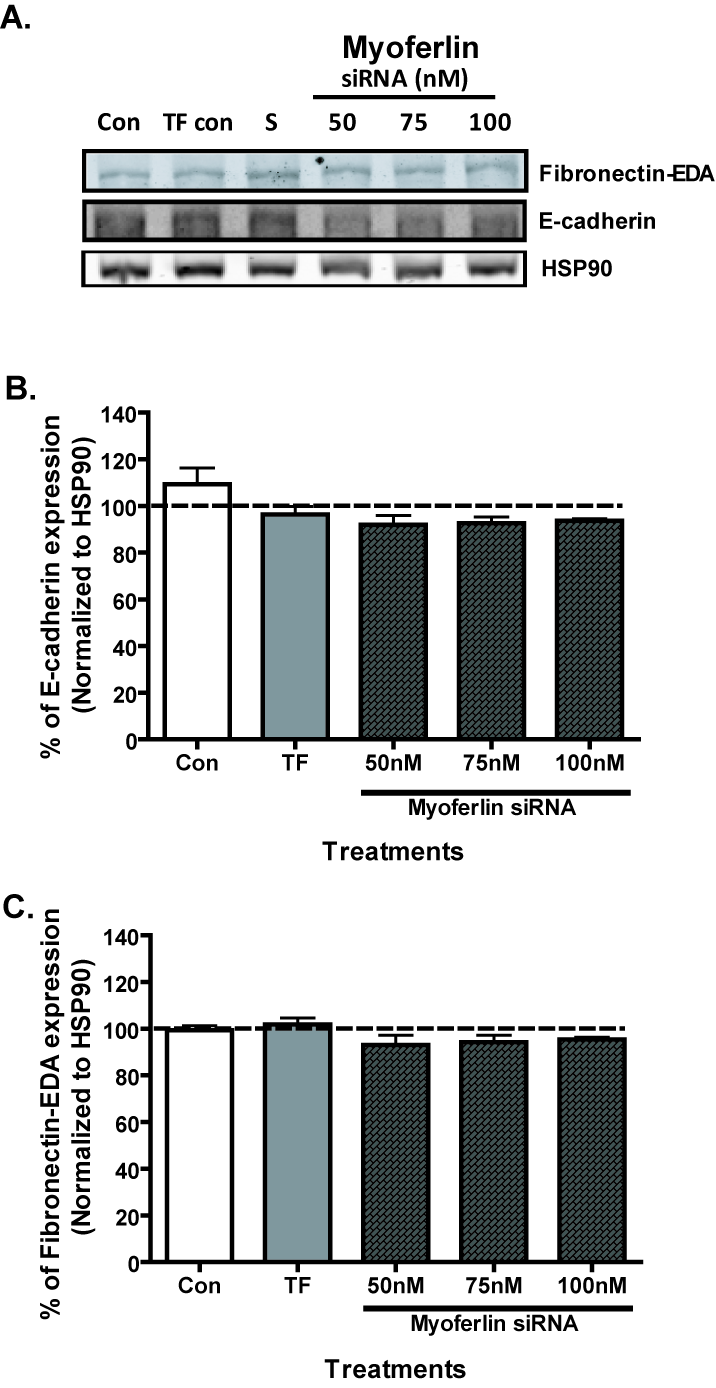

Supplement: Figure S2 — Myoferlin knockdown does not induce features of epithelial-mesenchymal transition. 16HBE cells were treated with control media (con), transfection control (TF con), scrambled siRNA (S), and (A) myoferlin siRNA at 50 nM, 75 nM and 100 nM for 72 hours. Total cell lysates were prepared to analyze the expressions of adherens junction molecule E-cadherin and fibronectin-EDA as demonstrated by the representative immuno blot from three independent experiments. HSP90 served as a loading control. (B) Densitometry analysis of band intensities for E-cadherin and (C) fibronectin-EDA normalized to HSP90 compared to the scrambled siRNA control (dotted line). Values are represented as mean (± SEM) of three independent experiments. (TIF) [file pone.0040478.s002.tif]
